# Histopathological Features of Symptomatic and Asymptomatic Honeybees Naturally Infected by Deformed Wing Virus

**DOI:** 10.3390/pathogens10070874

**Published:** 2021-07-10

**Authors:** Karen Power, Manuela Martano, Gennaro Altamura, Nadia Piscopo, Paola Maiolino

**Affiliations:** Department of Veterinary Medicine and Animal Productions, University of Naples “Federico II”, Via F. Delpino 1, 80137 Naples, Italy; manuela.martano@unina.it (M.M.); gennaro.altamura@unina.it (G.A.); nadia.piscopo@unina.it (N.P.); paola.maiolino@unina.it (P.M.)

**Keywords:** deformed wing virus, hypopharyngeal glands, flight muscles, honeybee immunity, honeybee pathology

## Abstract

Deformed wing virus (DWV) is capable of infecting honeybees at every stage of development causing symptomatic and asymptomatic infections. To date, very little is known about the histopathological lesions caused by the virus. Therefore, 40 honeybee samples were randomly collected from a naturally DWV infected hive and subjected to anatomopathological examination to discriminate between symptomatic (29) and asymptomatic (11) honeybees. Subsequently, 15 honeybee samples were frozen at −80° and analyzed by PCR and RTqPCR to determinate the presence/absence of the virus and the relative viral load, while 25 honeybee samples were analyzed by histopathological techniques. Biomolecular results showed a fragment of the expected size (69bp) of DWV in all samples and the viral load was higher in symptomatic honeybees compared to the asymptomatic group. Histopathological results showed degenerative alterations of the hypopharyngeal glands (19/25) and flight muscles (6/25) in symptomatic samples while 4/25 asymptomatic samples showed an inflammatory response in the midgut and the hemocele. Results suggest a possible pathogenic action of DWV in both symptomatic and asymptomatic honeybees, and a role of the immune response in keeping under control the virus in asymptomatic individuals.

## 1. Introduction

Among the factors that threaten the health and wellbeing of honeybees, a noteworthy variety of pathogens such as bacteria, viruses, fungi and parasites are mentioned. In recent decades, honeybee viruses have been studied for their potential impact on beekeeping productions, acquiring more and more importance in the research world. Viruses in honeybees were first described in 1913 [1] when an American researcher attributed to a virus the “sac” appearance showed by some diseased larvae, although the causative agent (Sacbrood virus) was not characterized until 1964 [2]. To date, at least 22 viruses that can infect honeybees have been described. Most investigated hives are found to be infected by at least one virus, but often multiple viruses are detected in one hive [3,4,5].

In addition to causing high economic losses, viruses negatively affect the morphology, physiology, and behavior of honeybees and, although individuals do not always show clinical signs, they are frequently associated with weakening and colony collapse [6]. Depending on the different pathways of infection and on the health status of the colonies, viruses can cause symptomatic infections, i.e., overt or clinical, and asymptomatic infections, i.e., covert or subclinical [7]. Symptomatic infections are characterized by clinical signs and by high levels of viral particle production, to which the insect either succumbs or survives according to the status of the immune system.

These symptomatic infections can be further divided into acute and chronic: the acute involve the active replication of the virus, with a high titer of viral particles in a short time and cause rapid death of the host with evident clinical signs. In extreme cases, when the production of high viral titers occurs during a short time, sudden death can occur without previous clinical signs (hyperacute infections) [8]. Chronic infections, on the other hand, imply a slow but constant production of viral particles during the life of the host, or during the duration of the infected life stage, with subsequent appearance of clinical signs. On the contrary, asymptomatic infections are characterized by persistence of the virus beyond life stage, vertical transmission and the absence of obvious symptoms, although there could still be a hidden cost for the host [8,9,10]. Asymptomatic infections can be latent and persistent [6]. In the first case the viral genome may be present as an extrachromosomal episome or may be integrated into the host genome with incomplete replication or no replication at all. In the second case, there is a constant but low production of viral particles in the host cells, and either the infected cell survives, or the limited number of dead cells is counterbalanced by the production of new cells. Persistent infections, therefore, represent a balance between host and persistent viral replication, where despite the infection, the host does not die. Moreover, asymptomatic infections can become symptomatic when the host homeostasis is unbalanced by stressors such as other pathologies, food deficiencies, and other environmental factors [9].

Deformed wing virus (DWV) is positive single-stranded RNA virus belonging to the genus Iflavirus, family Iflaviridae of the order Picornavirales [11] and is the most prevalent virus in honeybees, with a minimum average of 55% of apiaries infected across 32 countries [12]. The virus was first isolated in the 1982 in the UK by Bill Baley and Brenda Ball from dead Japanese honeybees showing particular deformity of wings [13]. Soon after, honeybees from the UK, Belize and South Africa died showing DWV symptoms. Ten years after, in the UK the virus was found in Varroa destructor-infested colonies, and it was then found in every location where *V. destructor* was well established [12]. Due to the link with *V. destructor* and following the huge spread of it around the world between 1970 and 1980, DWV altered its epidemiology and has currently a global distribution [14]. Except for Australia, Uganda and the Canadian island of Newfoundland, where the *V. destructor* mite has not been found, the presence of this particular virus has been reported in Africa, Asia, Europe, North America and South America [15]. DWV appears in three master variants DWV-A, DWV-B and DWV-C, plus numerous recombinations, often more virulent than the masters [16]. DWV-A was the first variant to be detected and it is closely associated to colony collapse [17]; however, DWV-B, previously termed Varroa Destructor virus-1, was found to be equally or more virulent than DWV-A when injected in high viral loads [18]. DWV-C was first described in U.K. honeybee samples from 2007 and linked in combination with DWV-A to the death of overwintering colonies [19].

Recent studies have shown that DWV is present in more than 64 species of insects and highly prevalent not only in honeybees, but also in more than 29 arthropod species associated with honeybee hives [12,20,21]. Within insects, DWV was found in bumblebee species *Bombus terrestris* and *Bombus pascuorum*, wasp species *Vespula vulgaris* and *Vespa crabro* and *Lasius* spp. ants [12,22,23], *besides A. mellifera*. DWV is a major pathogen of honeybees and its prevalence, strongly connected with the ectoparasite *V. destructor*, strongly increases honeybee colony mortality [24]. DWV is a low pathogenic virus that is capable of infecting all stages of development of honeybees, from eggs to adults, although it shows a higher replication in pupae [25,26]. It takes its name from the characteristic symptom that manifests itself in newly hatched honeybees with deformed or underdeveloped wings; these honeybees, unable to fly, can die shortly after emerging from the cell. Initially, the deformity of the wings had been attributed to the action of the *V. destructor* mite, as the symptom was more evident in conjunction with a strong infestation by the parasite [27]. Subsequently, DWV was identified as the etiological agent of wing deformity, emphasizing the association between the viral titers and the symptom [25,28]. However, although DWV is one of the few honeybee viruses to have its own characteristic clinical manifestation, it is known also to be present in apparently healthy colonies [10].

Although the pathology and virulence of DWV remain linked to horizontal vectored transmission by *V. destructor*, the presence of DWV has been demonstrated also in the absence of *V. destructor* [29]. Varroa-mediated virus transmission from adult honeybees to developing pupae is responsible for the display of the symptoms [24,30] such as early pupal death, deformed wings, shortened and swollen abdomen and discoloration of the cuticle in adult bees, and learning deficiencies [14]. Symptomatic DWV infection occurs primarily during autumn and in highly mite-infested colonies, where it constitutes predictive marker for winter colony losses [30,31]. According to the epidemiological model proposed by Chen et al. [9] two distinct moments of viral presence and infection can be recognized: in healthy and viable colonies, the virus remains latent / persistent without determining evident symptoms. Vice versa, in weak “stressed” colonies, the virus can abandon the state of latency, considerably replicate, increasing its virulence and causing the death of single individuals and depopulation of the colony. Among the main stressors identified in DWV infection, temperature decline could increase severity of viral infection in newly emerged honeybees (probably explaining the high levels of winter losses), while pesticides and poor nutrition could trigger the honeybee immune system making them more susceptible to viral infection, leading to colony collapse [32,33]. However, the main trigger for DWV symptomatic infection remains the uncontrolled Varroa infestation.

There is no doubt that the impact of viral diseases, especially DWV, in apiaries is a global threat to beekeeping and it is associated to honeybee colony loss [34]. Possible treatments against viral infections in honeybees are not known and legally recognized to date. Currently, a suitable treatment against Varroa is the best approach to fight DWV, since, after treatment, there is a gradual reduction of viral titers in colonies [35,36].

A deep knowledge of the crucial aspects of the viral pathogenicity, is important for realizing an effective control program, therefore, the aim of this preliminary study was to analyze any anatomo-histopathological findings in symptomatic and asymptomatic honeybees collected from a hive infected from DWV to try to better understand the pathological events underlying the infection.

## 2. Results

### 2.1. Anatomopathological Results

Anatomopathological examination confirmed the presence of alterations in 29/40 (72.5%) honeybees, namely deformed and crippled wings, discolored and shortened abdomens while 11/40 (27.5%) honeybees showed no lesions. Samples displaying alterations were classified as symptomatic (S) honeybees, while samples not showing anatomopathological alterations were classified as asymptomatic (A).

### 2.2. Biomolecular Results

**Figure 1:**
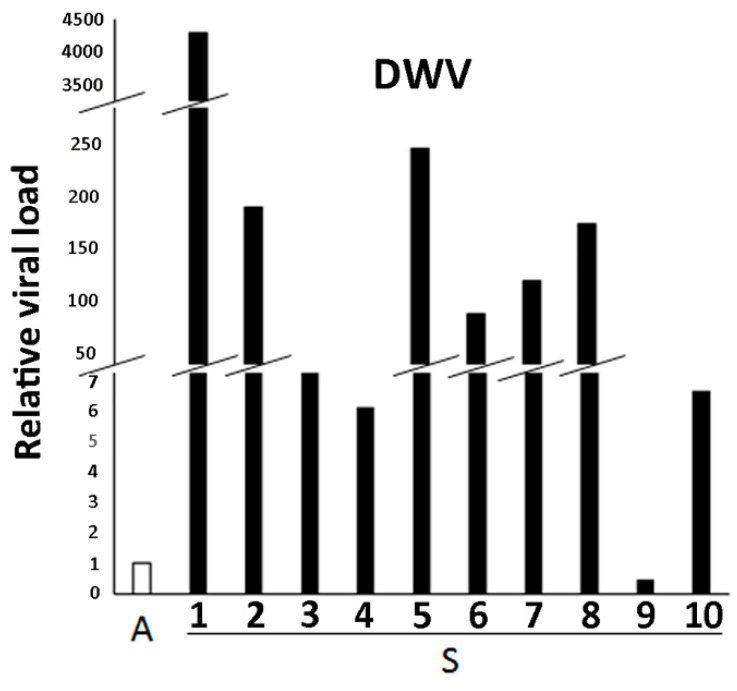
Analysis of relative viral load in honeybee samples showing clinical signs (S 1–10) compared with apparently healthy honeybee samples (A). Relative quantization data obtained by Real-time qPCR are expressed as fold change of each S sample with respect to the A samples considered as group (n = 5), which were set equal to 1, according to the 2^−ΔΔCq^ method.

A fragment of the expected size (69bp) of DWV was successfully amplified from 10/10 (100%) S samples and 5/5 (100%) A samples by RT-PCR but not in negative control (NTC) (data not shown). Act β amplification (151bp product) confirmed the integrity of all analyzed cDNAs. To gain insights on the possible difference of viral load between S honeybees and A group, samples were further investigated by qPCR. A successful and reproducible Cq of reference and target genes was obtained in 10/10 (100%) S and 5/5 (100%) A samples. RQ analysis according to 2^−ΔΔCq^ method revealed that the viral load was higher in 9/10 S samples (90%) compared to the A group (Figure 1). Further variant specific PCR analysis for identification of the DWV variant has revealed the presence of DWV-A but not DWV-B in all the 15/40 analyzed samples (data not shown).

Moreover, multiplex PCR for six honeybee viruses (Acute Bee Paralysis Virus-ABPV, Chronic Bee Paralysis Virus-CBPV, Sacbrood Virus-SBV, Black Queen Cell Virus-BQCV, Kashmir Bee Virus-KBV, Israeli Acute Paralysis Virus-IAPV) revealed the presence in all the 15/40 previously analyzed samples (symptomatic and asymptomatic honeybees) of ABPV (data not shown).

### 2.3. Histopathological Results

**Figure 2:**
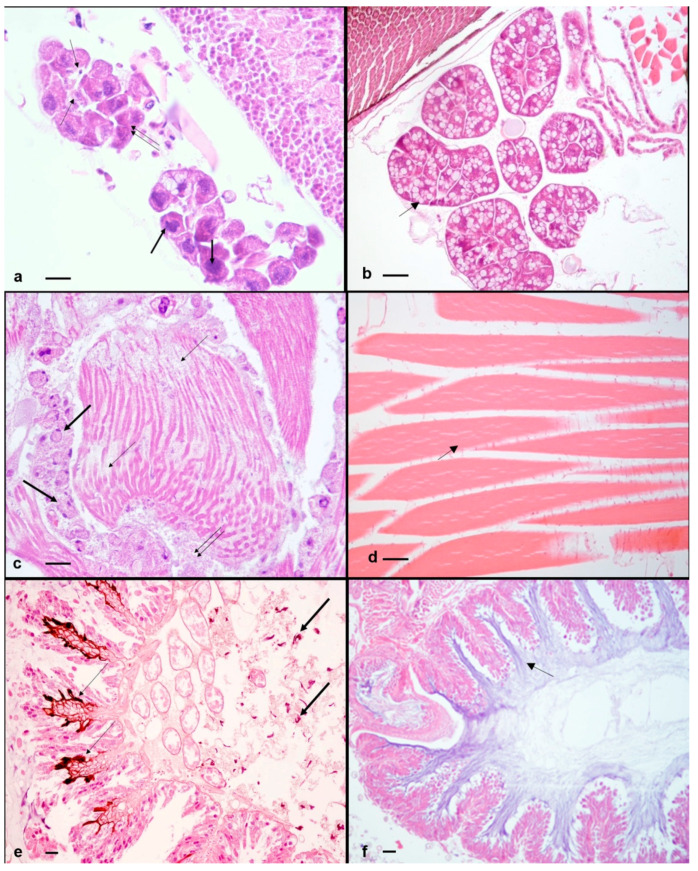
*A. mellifera* with symptomatic and asymptomatic infection of DWV and confirmed presence of ABPV. (**a**) Symptomatic honeybee. Hypopharyngeal glands. Small irregular acini showing cells with hyperchromic nuclei (thick arrows), cytoplasm filled with few small vacuoles and eosinophilic granules (double arrow), plasmatocytes in the gland lumen (thin arrows). H-E. 400× (40× objective and 10× ocular). (**b**) Asymptomatic honeybee. Hypopharyngeal glands. Large acini showing cells with cytoplasm filled with numerous large vacuoles with clear foamy material (thin arrow). H-E. 400×. (40× objective and 10× ocular). (**c**) Symptomatic honeybee. Flight muscles. Fibers with few not completely formed myofibrils (thin arrows), numerous muscle-forming nuclei (thick arrows), trophocytes with nuclear fragmentation and eosinophilic material between the muscle fibers (double arrow). H-E. 400× (40× objective and 10× ocular). (**d**) Asymptomatic honeybee. Flight muscles. Numerous fibers with many well-formed myofibrils (thin arrow). No trophocytes are present. H-E. 400× (40× objective and 10×ocular). (**e**) Asymptomatic honeybee. Midgut. Melanin accumulation between the fold of the villi (thin arrows) and in the hemocele (thick arrows). H-E. 200× (20× objective and 10× ocular). (**f**) Symptomatic honeybee. Midgut. Absence of melanization and hemocytes. The midgut epithelium appears intact and the peritrophic membrane appears well lined (thin arrow). H-E. 200× (20× objective and 10× ocular). Scale bar: 50 μm.

The histopathological analysis of symptomatic honeybees (19/25; 76%) revealed alterations of the hypopharyngeal glands in 19/19 (100%) honeybees and of flight muscles in 6/19 (31%) honeybees. The hypopharyngeal glands were characterized by small irregularly shaped acini, consisting of cells showing hyperchromic often fragmented nuclei and more or less abundant cytoplasm filled with few small vacuoles and numerous eosinophilic granules. Moreover, in the gland lumen and in the hemocele, it was noticed the presence of small cells with strongly basophilic nuclei and eosinophilic cytoplasm (Figure 2a).

**Figure 3:**
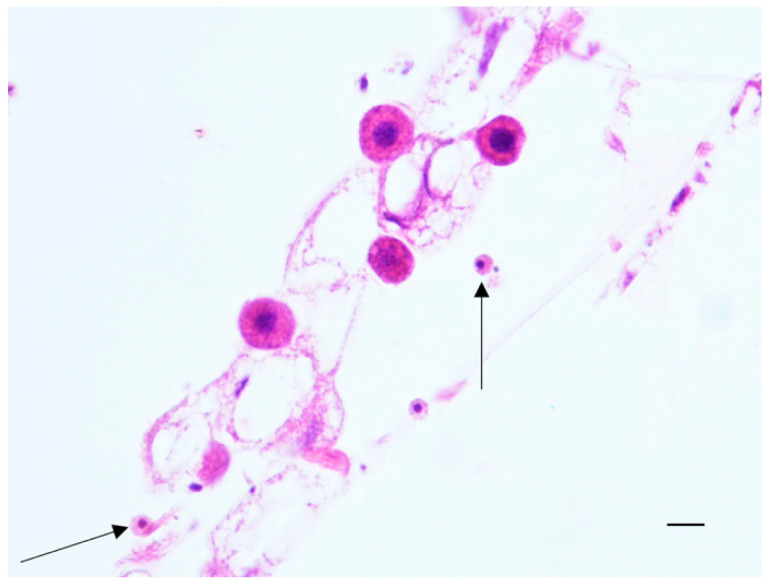
*A. mellifera* with asymptomatic infection of DWV and confirmed presence of ABPV. Fat Body. Granulocytes near the abdominal fat body (thin arrows). H-E. 400× (40× objective and 10× ocular). Scale bar: 40 μm.

In contrast, hypopharyngeal glands of asymptomatic honeybees appeared composed of larger acini, consisting of cells showing numerous large vacuoles with clear foamy material in the cytoplasm (Figure 2b). The flight muscles showed absence of tonofibrils, few myofibrils often not completely formed and many new muscle-forming nuclei indicative of an ongoing myogenesis and incomplete maturation. Moreover, trophocytes with nuclear fragmentation or absence of nuclei, intermingled with eosinophilic material were evident between the muscle fibers. (Figure 2c). In contrast, the flight muscles of asymptomatic honeybees consisted of numerous fibers showing many well-formed myofibrils. No trophocytes were observed in asymptomatic samples (Figure 2d). The histopathological evaluation of asymptomatic honeybees (6/25) highlighted the presence in 4/6 (66%) honeybee samples of a great amount of melanin between the folds of the villi and in the lumen of the midgut, and scattered in the hemocele (Figure 2e). Moreover, the presence of two cell populations (hemocytes) was observed: one population characterized by small cells, showing small and hyperchromic nuclei, often localized at the periphery, and clear, bright eosinophilic cytoplasm, identified as plasmatocytes; the second population characterized by bigger cells with dark nuclei and granular light eosinophilic cytoplasm, identified as granulocytes. Plasmatocytes were localized in the epithelium of the midgut and in the hemocele; granulocytes were mainly present near the abdominal fat body (Figure 3).

**Figure 4:**
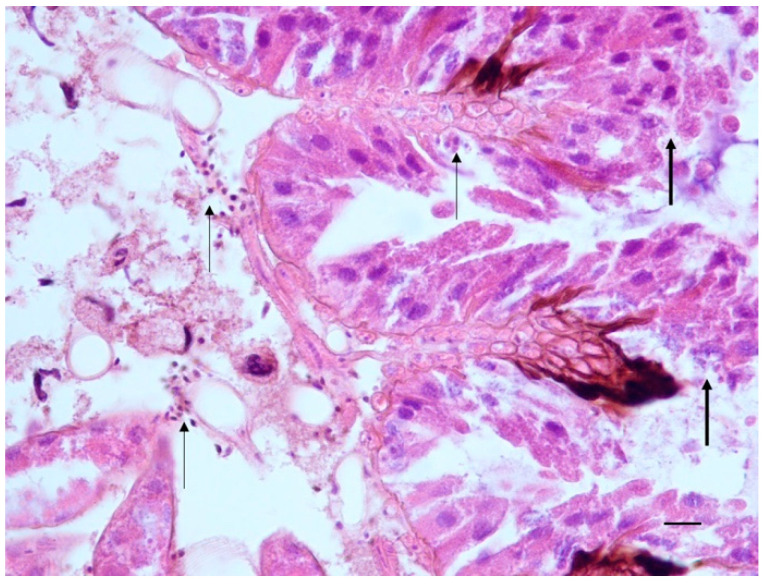
*A. mellifera* with asymptomatic infection of DWV and confirmed presence of ABPV. Midgut. Plasmatocytes in the epithelium and in the hemocele (thin arrows), epithelium exfoliation and disruption of the villi (thick arrows). H-E. 400× (40× objective and 10× ocular). Scale bar: 40 μm.

Where melanin deposition occurred at the basal lamina level of the midgut villi and high infiltration of plasmatocytes was present at this level, high level of midgut epithelial cell exfoliation and only few regenerative cell nests were observed; in the severest cases, epithelial cells showed pyknotic nuclei, and disruption of whole villi was noticed (Figure 4). On the contrary, in symptomatic honeybees melanization was not present and hemocytes were not observed in the midgut neither in the hemocele. The midgut epithelium appeared intact and the peritrophic membrane appeared well lined (Figure 2f).

Moreover, no spores of Nosema spp. were observed in any of the 25/40 analyzed samples.

## 3. Discussion

DWV is recognized, in association with *V*. *destructor*, as one of the main causes of colony collapse.

Unlike many other viruses, it is characterized by typical symptomatic infections showing high pupal mortality, wing deformities, shortened, bloated and discolored abdomens; however, the virus is also capable of infecting the entire colony silently [10,37]. Therefore, the scarce presence or absence of clinical signs may not reflect the actual state of health of the colony.

In this study, symptomatic and asymptomatic honeybee samples were collected and subjected to biomolecular analysis to highlight the presence of viral genome and to anatomo-histopathological analysis to evaluate the presence of any alterations of organs and tissues. Biomolecular results showed elevated DWV viral titers in S samples compared to A samples. Despite the limited number of samples analyzed, the results obtained agree with previous studies [10,35]. We can therefore imply that also the samples used for histopathological had high viral titers in symptomatic honeybees and lower viral titers in asymptomatic honeybees.

Honeybees exhibiting anatomopathological alterations showed also histopathological alterations of the hypopharyngeal glands and of the flight muscles.

In *A. mellifera* the hypopharyngeal glands are part of the digestive system and, according to the role played in the colony, they are responsible for the production of royal jelly, storage of glycogen for the flight muscles, synthesis of enzymes important for the transformation of nectar into honey and for social immunity [38,39]. Moreover, the presence of vitellogenin, a glycoprotein necessary to produce immune system components and for longevity, has been demonstrated in the hypopharyngeal glands [40]. In this study the hypopharyngeal glands of symptomatic honeybees appeared hypotrophic, containing few small vacuoles (mucous origin) and numerous eosinophilic granules (serous origin). This seems to suggest an alteration of the secretory activity, particularly a shift towards an increase production of serous secretion, typical of foragers [41,42]. A possible early passage of honeybees to their role as foragers could be responsible for an unbalance in the castes and premature aging of the colony. Considering the role of hypopharyngeal glands in producing components of worker and royal jelly, essential for the efficient development of larvae [43,44], a modification in secretion, could lead to an altered production of the components of this substance and a consequent altered development of the larvae, which could be weaker and more susceptible to the action of the virus and of other pathogens [45,46]. Moreover, it can be hypothesized that alterations of the hypopharyngeal glands could also lead to a reduced secretion of vitellogenin, and a consequent, at least partial, impairment of the immune system [47,48]. These effects, in the long run could compromise colony fitness and survival. Alterations of hypopharyngeal and mandibular glands of honeybees infected with DWV, have already been described by Koziy et al. [49] and our observations match what previously found, corroborating the theory of an action of the virus on these organs.

At the thoracic level, symptomatic honeybees showed incomplete development of the flight muscles. In healthy honeybees, the mature muscles begin to form during pupal development by replacement of the larval muscles with mature muscles, starting from new muscle nuclei with an end-to-end trend. At the same time there is a gradual reduction of the fat body due to the degeneration of the trophocytes. The myogenesis process ends 70 h after cell capping with the attachment of the muscles to the epidermis of the cuticle using tonofibrils [50]. The histopathological study of the flight muscles of symptomatic honeybees has highlighted the presence of eosinophilic material and trophocytic nuclear debris between the muscle fibers, most of which appeared immature and detached from the cuticle, consequent to the absence of tonofibrils. These aspects, found in adult honeybees, could be indicative of incomplete myogenesis and could be responsible of an altered development of honeybees and of a reduction of their size and inability to hatch and fly. Localization of DWV in the flight muscles of symptomatic honeybees was described by Lamp et al. [51], using immunohistochemical techniques, and we here describe for the first time the presence of lesions at this level. Additionally, in this study not all samples showed incomplete myogenesis, and the reason could be found in the different developmental moment in which the virus infects the honeybee or in the titer of the virus.

Interestingly, honeybees showing no anatomopathological alterations, despite being infected by the virus, did not show the same tissue alterations as the symptomatic ones, but revealed the presence of a high number of inflammatory cells (plasmatocytes and granulocytes) and melanin accumulation between the midgut villi and in the hemocele. These findings suggest a strong activation of the immune system, particularly of the cellular response. Honeybees can try to keep the virus under control thanks to an efficient individual immune system, which is mainly composed by a first line defense and a second line defense. Honeybee venom is present on the cuticle of adult honeybee and can be considered as a chemical barrier and a first line defense against pathogens in the individual [52]. The exoskeleton cuticle and the peritrophic membranes of the digestive tract, also are considered as a first line defense as they prevent pathogens from entering the body and have access to the cells [53]. If unfortunately, a pathogen manages to surpass these physical barrier, cellular and humoral immune responses will be activated as a second line of defense [54]. The cellular response consists in activation of hemocytes function including phagocytosis, nodulation, encapsulation of the pathogen, what in pathology is defined as “granulomatosis reaction”, and melanization [55]. The humoral response involves secretion of antimicrobial peptides (AMP), and other effectors, melanization, and the enzymatic degradation of pathogens by different pathways [54]. Richardson et al. [56] have identified and described the presence of two predominant cell types involved in the cellular response: granulocytes and plasmatocytes. Granulocytes exhibit a strong propensity for phagocytosis while plasmatocytes are involved in the encapsulation activity [57]. A strong and efficient immune system is the key for honeybee health and colony fitness.

In our study, the midgut epithelium of asymptomatic honeybees showed slugged epithelial cells, and as only few regenerative cell nests were present the adequate turnover that could restore the non-functional epithelium was not guaranteed. It is intuitive that a midgut showing these alterations cannot be functional both in absorption of nutrients and secretion of substances useful for the wellbeing of the peritrophic membrane, and consequently of the honeybees. It seems evident that, although no symptoms are evident, the virus is still acting on cells and tissues and that the activation of the immune response comes with a cost for the host.

This study has highlighted the presence of significant morphological alterations in symptomatic and asymptomatic honeybees infected with DWV and the results could suggest a possible pathological action of the virus in both groups of honeybees, and a possible role of the immune system, particularly of the cellular response, in keeping under control the virus in asymptomatic infections. It could be discussed that the alterations found could be linked to the action of other pathogens such other viruses or Nosema spp. However, histopathological examination of the midgut has been proven to be an efficient diagnostic tool for identifying the parasite in honeybees [58,59] and, as no spores have been observed in our samples, we can exclude the role of the parasite in generating the lesions observed. Regarding the possible action of other viruses, in this study we have screened for the presence of six different viruses and ABPV was found in all samples. ABPV is often associated to DWV in honeybee colonies [60], yet ABPV alone does not trigger humoral or cellular immune response in honeybees and therefore should not be considered as directly responsible for generating the immune response and melanization observed in the midgut and in the hemocele [61]. However, we cannot exclude a co-participation of ABPV to the generation of the alterations here found.

Therefore, further studies using other techniques such as FISH and immunohistochemistry, are necessary to deepen this preliminary study and better understand the etiopathogenesis of the findings here described.

## 4. Materials and Methods

During a regular visit to a beehive at an apiary located in Naples, Campania Region, it was possible to observe the presence of numerous small honeybees with deformed wings and shortened and discolored abdomens, suggesting the presence of a DWV infection.

The infected hive was clinically inspected, and the levels of *V. destructor* infestation were evaluated using the icing sugar technique [62] and assessed at 6% (18 mites/300 honeybees). A total of 40 adult honeybee samples were randomly captured from the frames and transported in 50 mL tubes to the laboratory of Veterinary General Pathology and Anatomical Pathology of the Department of Veterinary Medicine and Animal Productions, University of Naples “Federico II”.

### 4.1. Anatomopathological Analysis

After immobilization with chilling for 3 min at −20 °C [63], all collected samples (40) were observed at the stereo microscope (Microscope Axioskop HBO50, Zeiss, Milan, Italy) to better identify any anatomopathological lesions and classify individuals in symptomatic and asymptomatic according to the presence/absence of typical clinical signs of the disease.

### 4.2. RNA Extraction, Reverse Transcription (RT) and PCR

A total of 15/40 honeybees were subjected to biomolecular investigation to verify and, in case of positive results, quantify the presence of viral RNA.

Samples were individually chopped up with a sterile blade to facilitate subsequent homogenization with the TissueLyser mechanical homogenizer (Qiagen, Hilden, Germany). Each sample was put in 2 mL tubes along with a grinding metal bead and subjected to lysis by two steps of five minutes at 50 Hz, interspersed with a cycle of ice cooling of 2 min to avoid overheating and preserve the integrity of the biological molecules.

RNA was extracted and purified from genomic DNA using the RNeasy Plus Mini Kit (Qiagen, Hilden, Germany), according to the protocol provided by the manufacturer, and RNA concentration was measured by spectrophotometric reading.

For each sample, 250 ng of RNA were subjected to RT using the commercial iScript cDNA Synthesis Kit (Bio-Rad Laboratories, Hercules, CA, USA), according to the manufacturer’s recommendations.

Subsequently, 12.5 ng of cDNA for each sample were subjected to PCR to amplify a segment of DWV genetic material and verify the presence/absence of the virus in the samples using the AmpliTaq Gold DNA Polymerase kit (Applied Biosystems, ThermoFisher Scientific, Waltham, MA, USA) according to manufacturer’s instructions. The housekeeping gene β-actin (Act β) of A. mellifera was also amplified to ensure the presence of amplifiable cDNA in each sample. One no template control (NTC) was included in each PCR reaction as negative control.

Subsequently, a new PCR was performed on the same samples to discriminate between the two different variants DWV-A and DWV-B according to the protocols found in the literature [64,65].

Moreover, a multiplex PCR was executed on the previous samples (15/40) to screen for the presence of six other relevant honeybee viruses (ABPV, CBPV, SBV, BQCV, KBV, IAPV) according to the protocol proposed and validated by Cagirgan and Yazici [66]. The set of primers used for amplification of the genetic material of viruses and Act β used in this study were found in literature and a complete list, together with the product size, annealing temperature and application is reported in Supplementary Materials.

Amplification products were migrated by electrophoresis on 2.5% agarose gel in TBE buffer (Tris-Borate-EDTA) along with a 50 bp molecular marker (Bioline), stained with ethidium bromide and observed under UV with the ChemiDoc gel scanner (Bio-Rad).

### 4.3. Real-Time PCR (qPCR) for Detection of Relative Viral Load

In order to determine a relative quantization (RQ) of viral load of the samples, a Real-Time PCR (qPCR) was carried out using the primers described above.

For each sample tested positive for DWV in PCR, 12.5 ng of cDNA were subjected to qPCR using iTaq Universal SYBR Green Supermix kit (Bio-Rad), according to the manufacturer’s instructions.

Amplification of honeybee Act β as reference gene was also performed in parallel to allow normalization of the results and an NTC was included in the reaction as negative control.

Relative quantization of DWV viral load was calculated by using the 2^−ΔΔCq^ method as previously described [67,68]. Briefly, fold change in viral load was estimated for each individual S sample against A samples considered as control group.

### 4.4. Histopathological Analysis

Samples were processed as previously described [69]. Briefly, honeybees were individually injected with 10 μL of 10% buffered formalin and then stored for 24 h in 50 mL tubes containing the same fixative.

Subsequently, each sample was placed in an embedding cassette and processed. 3 μm sections were cut, stained with hematoxylin and eosin, and observed by light microscopy (Microscope Nikon Eclipse E-600, Tokyo, Japan). All tissues were observed to identify possible alterations and analyzed for the presence of visible pathogens, i.e., Nosema spp.

## Data Availability

Data are available on reasonable request to the corresponding author.

## Supplementary Materials

The following are available online at https://www.mdpi.com/article/10.3390/pathogens10070874/s1, File S1: Oligonucleotides used for amplification of viruses and Act β in this study. Sequences, products size, annealing temperature and applications are indicated.
