# Supplementary material for: Histopathological Features of Symptomatic and Asymptomatic Honeybees Naturally Infected by Deformed Wing Virus
Source: Pathogens. 2021 Jul 10;10(7):874. doi: 10.3390/pathogens10070874 (PMC8308782; doi:10.3390/pathogens10070874)
Supplement: Supplementary file 1 [file pathogens-10-00874-s001.zip › pathogens-1259044-supplementary.pdf]

Primers 5′→ 3′	Product size (bp)	Annealing T°	Application
DWV-F (5- GGATGTTATCTCCTGCGTGGAA -3) *<br>DWV-R (5-CCTCATTAACTGTGTCGTTGATAATTG -3) *	69	60	PCR/qPCR
Actβ -F (5- ATGCCAACACTGTCCTTTCTGG -3) **<br>Actβ -R (5- GACCCACCAATCCATACGGA -3) **	152	60	PCR/qPCR
DWV-A-F (5- GCGTGTTGCAACTCGCTTC-3)***<br>DWV-A-R (5-TGCCTGCACCGGATTCGATAAT-3)***	211	58	PCR
DWV-B-F (5-GCAAGTTGGAGATAATTGTA-3)****<br>DWV-B-R (5-CGATACTTACATTCTTCAAGAT-3)****	116	58	PCR
IAPV-F (5-CGAACTTGGTGACTTGAAG-3)*****<br>IAPV-R (5-GCATCAGTCGTCTTCCAGG-3)*****	110	58	Multiplex PCR
SBV-F (5-CGTAATTGCGGAGTGGAAAGATT-3)******<br>SBV-R (5-AGATTCCTTCGAGGGTACCTCATC-3)******	342	58	Multiplex PCR
AIV-F (5-GGTGCCCTATTTAGGGTGAGGA-3)******<br>ABPV-R (5-ACTACAGAAGGCAATGTCCAAGA-3)******	460	58	Multiplex PCR
BQCV-F (5-CTTTATCGAGGAGGAGTTCGAGT-3)******<br>BQCV-R (5-GCAATAGATAAAGTGAGCCCTCC-3)******	536	58	Multiplex PCR
AIV-F (5-GGTGCCCTATTTAGGGTGAGGA-3)*******<br>KBV-R (5-TGCACGGGAAGTATAAATAATTCT-3)*******	641	58	Multiplex PCR
CBPV-F (5-AACCTGCCTCAACACAGGCAAC-3)******<br>CBPV-R (5-ACATCTCTTCTTCGGTGTCAGCC-3)******	774	58	Multiplex PCR

Oligonucleotids used for amplification of viruses and Act β in this study. Sequences, products size, annealing temperature and applications are indicated. F:Forward; R: Reverse; DWV:Deformed Wing Virus; Actβ:Actin β; DWV-A:Deformed Wing Virus-A variant; DWV-B:Deformed Wing Virus-B variant; IAPV:Israeli Acute Paralysis Virus; SBV:Sacbrood Virus; AIV:Apis Iridescent Virus; ABPV:Acute Bee Paralysis Virus; BQCV:Black Queen Cell Virus; KBV: Kashmir Bee Virus; CBPV:Chronic Bee Paralysis Virus

*70;**71;***64;****65;*****72;******73;*******74
